# Editorial: With Obesity Becoming the New Normal, What Should We Do?

**DOI:** 10.3389/fendo.2019.00250

**Published:** 2019-04-17

**Authors:** Katherine Samaras, Hendrik Tevaearai, Michel Goldman, Johannes le Coutre, Jeff M. P. Holly

**Affiliations:** ^1^Department of Endocrinology, St Vincent's Hospital, Darlinghurst, NSW, Australia; ^2^Diabetes and Metabolism, Garvan Institute of Medical Research, Darlinghurst, NSW, Australia; ^3^St Vincent's Hospital, St Vincent's Clinical School, Darlinghurst, NSW, Australia; ^4^Department of Cardiovascular Surgery, Inselspital, Bern University Hospital and University of Bern, Bern, Switzerland; ^5^Institute for Interdisciplinary Innovation in Healthcare, Universite Libre de Bruxelles, Bruxelles, Belgium; ^6^Department of Medicine, Imperial College London, London, United Kingdom; ^7^Nestlé Research, Lausanne, Switzerland; ^8^Professor of Clinical Science, IGFs and Metabolic Endocrinology Group, Translational Health Sciences, Faculty of Health Sciences, Bristol Medical School, University of Bristol, Bristol, United Kingdom

**Keywords:** obesity, global health, public health, epidemic, childhood obesity, weight loss, polycystic ovary syndrome, non-alcoholic liver disease (NAFLD)

In just a blink oft Earth's eye (approximately three decades), obesity has become a global epidemic and an urgent health crisis due to its impact on health services and the loss of human capital. It is not just a crisis for health professionals, health economists, and government officials managing finite resources and considering the economics of premature loss of life and economic productivity: it is a major societal concern that challenges the way we think about and manage resources. These resources can be considered in terms of health, human, budgetary, financial, and primary products. In this timely Frontiers Research Topic, researchers from a breadth of disciplines internationally contributed reviews, meta-analyses, and novel data on the challenges obesity presents in attempts to stimulate debate on ways forward.

The impact of modernity on body composition homeostasis is reviewed by Tremblay who challenges us to reconsider the paradigm that imbalances between food intake and physical activity are the only determinants of the obesity epidemic, given that complexities within the energy balance equation prevent a reductionist approach. Factors influencing or regulating the endocrine responses to energy-in and energy-out are discussed, including the impacts of contemporary lifestyle: alterations in the sleep-wake cycle, shortened sleep duration, environmental pollutants, high mental cognitive work, and stress. The challenge of weight loss maintenance in the face of an endocrine system that has evolved to defend body fat stores and promote weight regain is examined. The solution? In the face of the obesity pandemic, promotion of the healthiest lifestyle possible. We should not be limited by focusing solely on energy balance, but holistically intervene on sleep hygiene, reducing stress responses, and limiting environmental exposure to pollutants and endocrine disruptor chemicals, all with the aim of down-regulating the central-, adrenal-, and adipokine-regulating hormonal responses that mitigate an individual's hard won weight loss. Whilst government intervention is required to address some of these factors (such as creating safe environments), clinicians can play an important role in guiding patients to battle against the regulatory pathways and modernity factors that promote weight regain. With this in mind, clinicians must realize that the work is not done when weight loss is achieved: we keep our obese patients for our working life, as they will require ongoing guidance, supervision, and intervention.

Where governments have shown leadership in developing and implementing policies to address obesity, the presence of bias or influence is a significant concern. In an environment where everyone is an “expert” on diet and obesity [informed from diverse sources including media, talk-show hosts, reality and lifestyle programs, advertorials, internet blogs and (occasionally) real science], do obesity policy makers and service deliverers bring their own prejudices to the execution of professional duty? Pengilley and Kelly demonstrate that uninformed opinion does influence policy implementation. They recommend that development and design of obesity policy requires a strict, robust, and transparent governance framework to prevent these prejudices from derailing the efficacy, reach, and delivery of obesity policy. Their findings and comments are prophetic, published just months before The Lancet published evidence of undue influence by a multinational sweetened beverage corporation on obesity science and policy in China (1) and, separately, Milbank Quarterly published evidence of efforts by a multinational beverage corporation to influence the US Centers for Disease Control and Prevention (2). A strongly worded editorial in Lancet Oncology very recently stated that “Governments must not allow their public health strategies to be unduly influenced by powerful multinationals who might be more concerned with protecting their own interests than helping to solve this ongoing health crisis ” (3).

Where government has implemented population-based obesity prevention interventions, proof of cost-effectiveness is vital, given precious health resources. The paper from Döring et al. evaluated the cost-effectiveness of PRIMROSE, a program aimed at Swedish pre-school children that addressed healthy eating and physical activity, with the primary outcome of BMI at age 4. Cost-effectiveness was not established, due to the cost of educating nurses to implement the program and parental income lost to attend. These results raise a number of questions for intervention implementation (e.g., utilizing trained but less expensive staff). As obesity impacts on sick leave and reduced productivity at work, employers are understandably focused on their employees' health. Feldman et al. examined the effectiveness of a workplace wellness program in obese attendees and found that whilst the program was associated with only very modest weight loss, the weight loss was clinically meaningful in attendees who were ready for change. These findings assist in determining weight loss resource allocation for greatest impact. Thus, publicly and privately funded initiatives for obesity reduction are likely to make very little or only a modest dint in the obesity epidemic. The serious fundamental question in the modern epidemic of obesity is: does the solution lie in influencing the choices individuals make in an obesogenic environment (marketing works commercially, especially for foods), or is it altering or limiting the choices available to individuals, using legislation if the food industry insufficiently responds to the obesity crisis? Can we learn anything from the efficacy of government legislations that, for example, restricted the sale of alcohol (Sweden), highly taxed cigarettes (Ireland), or introduced plain packaging and graphic warnings on tobacco products (Australia)? (4). Each represented a government legislative response to harmful products and have reduced consumption and the adverse health outcomes associated with each product. Sugar taxes are hotly debated internationally. A recently published review showed that excise taxation of tobacco, alcohol, and sweetened beverage works in reducing consumption (5). Provocative food for thought.

Following on from the theme of childhood obesity prevention are three papers that report factors associated with childhood obesity and the surprising lack of cardiometabolic risk reduction with weight loss intervention. Corica et al. present sobering data in obese children aged 2–17 years, demonstrating high rates of obesity and cardiometabolic disease within the family; extreme youth did not protect against a deteriorated metabolic profile. Dalla Valle et al. similarly reported an almost universal presence of at least one cardiometabolic risk factor in obese children aged 2–17 years; obesity intervention did not improve these without a very substantive weight loss. When we consider obesity-accelerated diseases such as diabetes, heart disease, and cancer and the known consequent reduced life expectancy, these data indicate the urgent need for effective public health preventative measures, in addition to family-based interventions addressing intergenerational factors and treating not only the afflicted children, but also their parents.

Relevant to childhood obesity is consideration of the impact of obesity on reproductive health and the intrauterine environment, since excess maternal weight and weight gain in pregnancy adversely affects birth weight and subsequent childhood obesity risk. Obesity impacts the risk for polycystic ovary syndrome and reduces fertility and success of assisted reproduction. Additionally, maternal obesity impacts the risk of pre-eclampsia and gestational diabetes, unplanned Cesarean section and fetal and neonatal outcomes: not limited to premature delivery, neonatal hypoglycaemia, macrosomia, and childhood-onset of obesity. The impact of obesity on the efficacy and outcomes of assisted reproduction, reviewed by Tziomalos and Dinas examines these issues in polycystic ovary syndrome, including the evidence for weight reduction prior to assisted reproduction, recommending deferment of conception until a healthier weight is achieved.

When the impact of intrauterine over-nutrition and macrosomia on obesity risk in childhood are considered, a rationale for public health measures for healthy maternal weight throughout the reproductive years is justified. Farpour-Lambert et al. examined the evidence for lifestyle interventions during pregnancy and postpartum impacting maternal and neonatal outcomes, reporting that they reduce gestational weight gain, pregnancy-induced hypertension, the need for Cesarean section and neonatal respiratory distress syndrome, without any risk of harm to the mother or neonate. These benefits were observed across all BMI categories. Dietary interventions were associated with the greatest reduction of gestational diabetes, pregnancy-associated hypertension and preterm birth. The synthesis of the evidence reviewed substantiates that a low glycaemic load and 30–60 min of physical activity most days should become health policy directives for women during pregnancy and postpartum. In response to the Farour-Lambert paper, Skouteris et al. call attention to the lack of any international consensus guidelines on weight management pre-conception. The impact of weight status in the pre-conception phase on fertility, pregnancy and subsequent maternal and infant outcomes is reviewed. The importance of efforts to improve the lifestyle and weight status of mothers-to-be is highlighted, with calls for international efforts. Thus, healthy lifestyle and weight in the pre-conception period and during pregnancy are critical factors in assisting newborns get off to the best start as they enter our brave new obesogenic world.

Moving away from policy, politics, and lifestyle interventions, this Research Topic includes papers enlarging our scientific understanding of energy homeostasis and its links to other organ systems. A challenge to clinicians treating and researchers investigating people with obesity is predicting energy expenditure. In a large population of morbidly obese participants, Cancello et al. examined existing models for energy expenditure calculation against indirect calorimetry. They found most models inaccurate and recommended indirect calorimetry.

The fascinating interface of gut hormone regulation in response to nutrient intake and how these hormones regulate appetite and metabolism are reviewed by Hope et al. particularly in how these hormones respond to fasting, bariatric surgery and with pharmaceutical analogs. Future therapies are discussed; particular consideration is given to the co-targeting of multiple regulatory pathways in order to tackle the many different ways that human physiology has evolved to defend energy stores in the face of (potentially life-threatening) energy deficiency. Thus far, science is yet to detect a hormonal or chemical signal that effectively says: “stop eating, our adipose stores are full.” We are challenged to determine how to enhance the effects of the homeostatic effects of important gut hormones.

The central regulation of food intake is a key factor in obesity development: what makes some people say “enough” and other to say “more please” when faced with the same meal or more food? Over three decades, the number of players regulating the physiology of appetite and satiety has expanded rapidly resulting in a greater understanding of the role of numerous newly discovered gut peptides and the contribution of circadian and stress hormones. Lasschuijt et al. present novel data examining the impact of oro-sensory and central pathways involved in the endocrine response to food ingestion, specifically, the effects of the duration of chewing food and sweet taste intensity on satiety and endocrine responses. In a series of elegant experiments, healthy weight participants consumed isocaloric foods of differing textures and sweetness but with similar macronutrient composition followed by measurement of hormonal responses and subsequent food intake. Modest increases were found in pancreatic polypeptide following hard-sweet food ingestion. Interestingly, eating and spitting out the food was associated with higher acute insulin responses. These data suggest that hormonal responses are affected by food texture, not just sweetness or caloric content. The act of chewing impacts hormonal responses to food, even without ingestion. It is possible lingual sweet taste receptors may contribute, as we start to understand the unique endocrinology of the tongue. More research in this fascinating area is awaited.

Physicians and metabolic researchers are increasingly aware of the important role the liver plays, not only as a synthetic factory for essential proteins and glucose and a passive reservoir for excess lipid, but as a key regulator of many aspects of metabolism. It is not only an innocent bystander in the physical onslaught of obesity but also the victim of the inflammatory effects of chronic excessive nutrient intake. Obesity is now the commonest cause of chronic liver disease in countries spared the epidemic of hepatitis B. The widespread metabolic associates of the spectrum of non-alcoholic fatty liver disease (NAFLD) are currently being delineated. Tarantino et al. demonstrate associations between the presence of obesity-associated NAFLD and carotid artery intima media thickness, an index of atherosclerosis, apparently mediated by the degree of visceral adiposity, age, and haematocrit. These data add to our understanding in the clinical evaluation of obese patients.

Depression is closely linked to obesity. Ouakinin et al. review the evidence for neuroendocrine links between adipose tissue, the hypothalamus-pituitary-adrenal axis, the sympathetic nervous system, and circulating and tissue-based inflammation. The review provides a framework to deepen our understanding of how energy homeostasis, stress, and neuroendocrine responses are connected, to the degree that both obesity and depression share biological mechanisms and pathogenesis.

The search for blood proteins in the early detection of cancers is a promising area that may improve health outcomes. As obesity is a major contributor to the risk of many cancers, the discovery of novel or sentinel markers for early cancer detection is important. Xu et al. showed that serum zinc-α2-glycoprotein levels, a newly recognized adipokine, were higher in Chinese patients with colorectal cancer. Validation of this interesting finding in larger populations and evaluation of its efficacy as an early cancer biomarker is awaited.

Epigenetics is an expanding area of interest particularly in understanding how environmental factors up- or down-regulate genetic susceptibility to obesity. Zhu et al. review transcription factors implicated in obesity pathogenesis and mechanisms of epigenetic modification. This area presents a number of possible mechanisms by which future interventions may mitigate against the impact of environmental factors on obesity susceptibility. It represents an exciting area for future research.

The potential metabolic benefits for traditional Chinese medicine contribute to the pharmacotherapeutic armamentarium to battle obesity as reviewed by Huang et al. examining the evidence for some medicinal components to improve metabolism. Challenges in translating plant and animal-based extracts into traditional Western pharmaceuticals are discussed. Many of our patients purchase supplements with claims of benefit in an unregulated, frequently not evidence-based, but highly profitable, supplements environment. Clinicians who are well-informed on the evidence for supplements of traditional Chinese medicine are best placed to advise their patients on where there may or may not benefit.

As editors of this Research Topic, we invited authors to examine the challenges we face in the broad areas that obesity impacts. We were rewarded by high quality contributions across the breadth of medicine, clinical, and fundamental research and public health. We anticipate the reader is informed by this collection of articles that embrace the many challenges obesity holds for all of us in basic and clinical science, as clinicians, public health planners, policy makers and consumers. To answer the question posed by this Research Topic: what should those of us in the health sciences do? Our mandate is clear, as obesity will erode all the progress we have made in human health over the last 100 years. First, we require a huge skilled health industry workforce and physical resources to tackle the health issues that people with obesity experience today and into the immediate future: we must train scientists, doctors, and allied health professionals to effectively treat, alleviate or palliate those suffering from obesity today and tomorrow. Second, our governments and industries need to be provided with evidence, so that they understand they will require deep pockets to support the immense loss of human and health capital that is occurring now and will do so for the immediate future, impacting economies internationally. Third, we must lobby for leadership from forward-looking and enterprising food industries and corporations to create products for harm minimization: food of lower energy content, smaller portion size, foods that promote satiety not hunger, an ethos of not promoting over-consumption. We must encourage food industry “disruptors”: enterprising food producers and manufacturers ready to exploit the opportunities presented in a market of consumers demanding better foods, when “old school” food industry doesn't respond with real change (only cosmetic or cynical change). Finally, we suggest that our policy makers and politicians should respond with taxation, levies, and laws that protect one of the most valued resources our society has: human health. Many governments have done so with other toxic substances, such as cigarettes and alcohol, occupational and environmental hazards. Such legislations are present already in some countries and cities, with sugar taxation and portion size limits: these could be extended elsewhere. Imagine a world however, where these measures were not required. Imagine a world where food is nourishing and beneficial, not harmful.

## Author Contributions

KS drafted this editorial. HT, MG, JlC and JMPH each contributed by revising and editing.

### Conflict of Interest Statement

The authors declare that the research was conducted in the absence of any commercial or financial relationships that could be construed as a potential conflict of interest.
